# IDEAL–compliant implementation of the Dexter^®^ surgical robot in cholecystectomy: a comprehensive framework and clinical outcomes

**DOI:** 10.1515/iss-2024-0033

**Published:** 2024-12-30

**Authors:** Jonas Dohmen, Julia Weber, Jan Arensmeyer, Philipp Feodorovici, Jonas Henn, Joachim Schmidt, Jörg C. Kalff, Hanno Matthaei

**Affiliations:** Department of Surgery, University of Bonn, Bonn, Germany; Bonn Surgical Technology Center (BOSTER), University of Bonn, Bonn, Germany

**Keywords:** dexter surgical robot, cholecystectomy, robotic surgery, IDEAL framework, implementation, patient safety

## Abstract

**Objectives:**

The integration of advanced technologies is transforming surgical practice, particularly through robotic systems. This study presents the early clinical implementation of the Dexter^®^ surgical robot for cholecystectomy and evaluates clinical outcomes using the IDEAL framework.

**Methods:**

Twenty patients underwent elective robotic-assisted cholecystectomy using the Dexter^®^ robot. A thorough implementation process, including rigorous surgeon and nurse training and standardized care protocols, was established. Data on operative metrics, complications, and patient outcomes were analyzed, and patient well-being was assessed via a postoperative phone survey.

**Results:**

Six surgeons and thirty nurses were trained, with surgeons completing a minimum of 20 h of simulation. Preoperative and operative times were significantly reduced through this process. Comparing the first 10 operations to the second, docking time decreased from 11.4 ± 4.1 min to 7.1 ± 2.1 min (p=0.0144) and operative time improved from 130.5 ± 25.7 min to 99.7 ± 21.8 min (p=0.0134). Mean intraoperative blood loss was minimal, averaging 19.5 ± 31.4 mL, and the average length of hospital stay was 3.1 ± 1.4 days. Postoperative pain levels were low, and patient satisfaction was high, as assessed by telephone survey.

**Conclusions:**

Our findings highlight the value of the IDEAL framework in guiding the systematic evaluation and implementation of new surgical technologies such as the Dexter^®^ robot. A structured approach is essential to improve patient outcomes and safety in the coming digital transformation of surgery.

## Introduction

The surgical field is experiencing a paradigm shift with the advent of advanced technologies [1], demanding careful consideration and strategic implementation [2]. Operating rooms are complex environments where precision, safety, and efficiency are paramount. Despite the rapid development of surgical robots [3], [4], [5], [6], a comprehensive implementation framework for their integration into clinical settings is yet to be fully established. Such a framework is essential to maximize the benefits of these technologies while ensuring patient safety and procedural efficacy [7].

Since the emergence of robotic surgery, telemanipulation systems have revolutionized surgical practice [8], [9], [10]. Over the years, robotic technologies have proven to offer significant advantages, primarily through the benefits of improved dexterity and precision [11], 12]. However, successful implementation requires meticulous planning, extensive training, and standardized protocols [7]. The integration process must address both technical and organizational challenges to ensure seamless operation.

The IDEAL (Idea, Development, Exploration, Assessment, and Long-term study) framework was established to provide a structured approach to the evaluation and implementation of surgical innovations [7], 13]. The framework emphasizes stepwise development and rigorous assessment, ensuring that new techniques and technologies are introduced safely and effectively. However, recent reviews have shown that the IDEAL framework is often not followed adequately in surgical research, highlighting the need for systematic tools to facilitate incremental evaluation and reporting of surgical innovations [14].

In our clinic, we developed a comprehensive implementation process based on the IDEAL framework, considering technical nuances and collaborative aspects for successful adoption. This study details our experience with 20 cholecystectomy procedures performed using the Dexter^®^ Robotic System by Distalmotion SA (Epalinges, Switzerland). We carefully followed all required steps for a modern implementation and achieved successful outcomes. Cholecystectomy, being a standardized, frequent, and relatively low-risk procedure, served as an ideal starting point for the implementation before expanding to other surgeries [15], 16].

Our procedure aims not only to present the current state of implementation of the Dexter robotic system, but more importantly to pave the way for future investigations, using the IDEAL steps that can be built upon in subsequent studies.

## Materials and methods

### IDEAL framework compliance

The IDEAL framework was used as a guideline to structure our implementation and reporting process. Our study primarily encompasses the development and exploration stages (Stage 2a and 2 b). The development stage involved pilot surgeries and the establishment of protocols, while the exploration stage focused on systematic implementation and data collection to assess outcomes. The IDEAL framework in relation to robotic cholecystectomies is shown in Table 1.

**Table 1:** Key factors for IDEAL framework compliance in robotic cholecystectomy.

IDEAL factor	Implementation details
Idea stage	Concept development, preliminary research, and identification of need for a new robotic system.
Development stage	Pilot surgeries, extensive training programs (including cadaveric and dry lab training), and initial protocol development.
Exploration stage	Systematic implementation, data collection on operative metrics, complications, patient outcomes, and iterative refinement of protocols.
Assessment stage	Interim analysis of initial results, assessment of clinical outcomes, patient satisfaction, and preparation for broader implementation.
Long-term study	Long-term follow-up of patient outcomes, cost-effectiveness analysis, and assessment of the sustainability and scalability of the robotic system.

### Patient selection

Patients with benign gallbladder disease (symptomatic cholecystolithiasis, presenting with symptoms such as biliary colic or biliary dyskinesia) and patients with imaging-confirmed gallstones or gallbladder polyps warranting elective surgical intervention were included in the study with some specific selection criteria to ensure the safety and efficacy of the robotic-assisted procedures. Cholecystectomies were planned as robotic procedures when patients consented to the procedure, the surgical robot was available, and the presence of Dexter^®^-experienced nursing and medical teams was confirmed. None of the intended patients declined a robotic intervention. Individuals below the age of 18, as well as those with morbid obesity (BMI>40), were excluded from the study. Additionally, cases requiring emergency surgery were not included. Patients presenting with contraindications identified during explorative laparoscopy, such as severe acute inflammation or anatomically unclear situations, were also excluded. Other common contraindications included severe comorbid conditions, such as advanced cardiovascular, pulmonary or liver diseases, were exclusion criteria, as these could increase perioperative anaethetic and surgical risk.

This selection ensured a homogenous patient population, facilitating a safer and more controlled implementation of the Dexter robot.

Prior to the procedure, all patients were Informed of the innovative nature of robotic cholecystectomy and provided with written consent. Institutional Review Board (IRB) approval was secured (IRB Number: 251/23–EP and 2024–147–BO). The implementation process and all operative procedures were conducted between March 2023 and August 2024 at the University of Bonn.

#### Dexter^®^ robotic system

The Dexter^®^ surgical robot represents a novel approach of robotic-assisted surgery, aligning with the concept of “on-demand robotics”. This technology enables surgeons to switch between robotic-assisted and laparoscopic surgery, as required [17], 18].

The system is equipped with two multi-jointed robotic arms that provide a greater range of motion compared to traditional laparoscopic instruments [19]. These robotic arms mimic the dexterity of the human hand, thus enabling precise movements during surgery. This flexibility allows the surgical team to utilize the advantages of both techniques, adapting the surgical approach based on intraoperative findings [20], 21]. The configuration of the surgical workspace is optimized to ensure minimal obstruction by the robotic arms, thereby facilitating enhanced access to the patient [22].

### Implementation process

**Figure 1:**
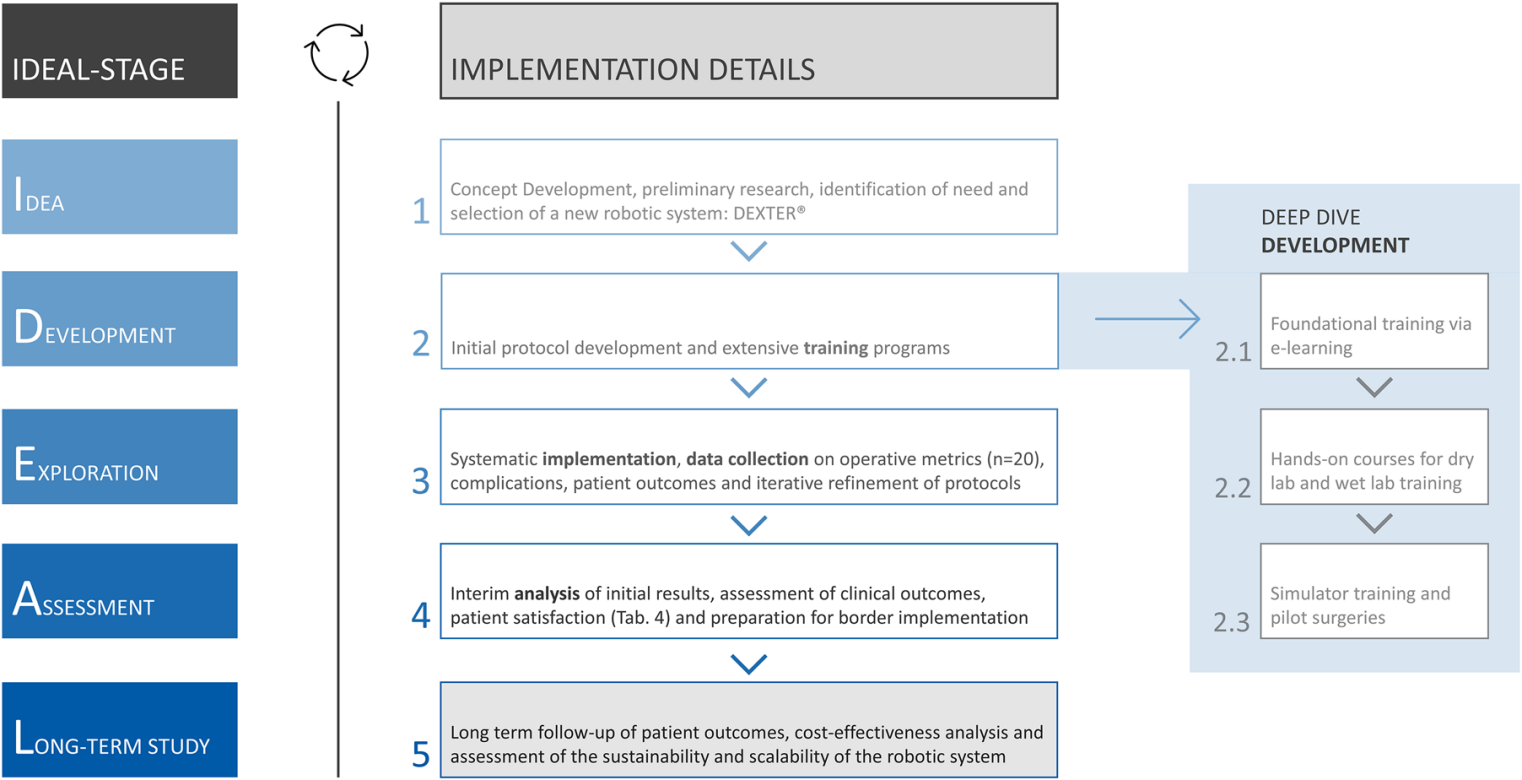
Flowchart of the comprehensive implementation process in compliance with the IDEAL framework, including training, workflow development and continuous monitoring stages.

**Figure 2:**
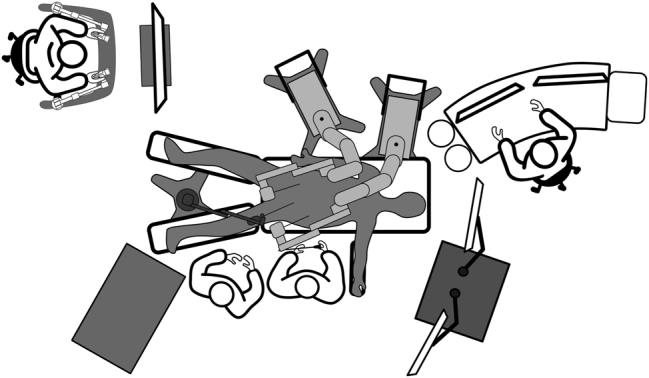
Dexter^®^ system and operative setup, illustrating the multi-jointed robotic arms and the arrangement within the operating room.

Implementing a new robotic system like the Dexter^®^ robot according to the IDEAL framework requires several key steps (Figure 1):

1. **Preparation and Training:** This involves extensive training for the entire surgical team to ensure proficiency and a uniform knowledge base among team members with the new technology.
  – **Surgeon Training:** Comprehensive training programs were developed, including didactic sessions, hands-on workshops, cadaveric training, and dry lab training over four months. Surgeons completed a minimum of 20 h of simulation training. One experienced HPB consultant (HM) with prior expertise in robotic surgery (Intuitive Da Vinci^®^) took part in all surgeries. A team of five surgeons, who completed the Dexter^®^ training program, participated in the operations on a rotating basis. The latter had experience as a first assistant in robotic surgery (Intuitive Da Vinci^®^). In order to be eligible to operate as the leading operating surgeon, they were obligated to previously acquire experience as a second surgeon in at least five operations. Two of these five surgeons reached this number during the study. At least one clinical specialist from Distalmotion SA attended every operation entirely during the implementation phase to provide additional support and ensure proper use of the robotic system.
  – **Nursing and Technical Staff Training:** Specialized training modules for operating room nurses and technical staff included both, theoretical knowledge and practical hands-on sessions to familiarize them with the robot’s setup, maintenance, and intraoperative assistance. Emphasis was placed on imparting knowledge related to patient setup, effective system handling, proper draping techniques, and dealing with emergency scenarios.
2. **Operational Workflow Development:** This involves creating and refining protocols to integrate the new technology smoothly into clinical practice.
  – **Workflow Design:** A detailed operational workflow was developed to integrate the Dexter robot into existing surgical protocols. This involved collaboration anesthesiology department to identify and address potential bottlenecks.
  – **Protocol Establishment:** Standardized protocols for preoperative planning, intraoperative management including emergency situations, and postoperative care were created, defining roles and responsibilities of each team member during the procedure.
3. **Trial Phase:** This involves conducting pilot surgeries to hone workflows based on real-world feedback.
  – **Pilot Surgeries:** Initial trial surgeries were conducted under controlled conditions with close monitoring. Feedback from these procedures was used to refine operating procedures. A standardized operative setup was established during this phase (Figure 2).
  – **Continuous Monitoring:** A robust monitoring system was implemented to collect data on operative metrics, complications, and outcomes. Regular multidisciplinary team meetings were conducted to discuss findings and implement necessary adjustments. Thorough documentation was conducted by study nurses and graduate students to ensure data accuracy.

**Figure 3:**
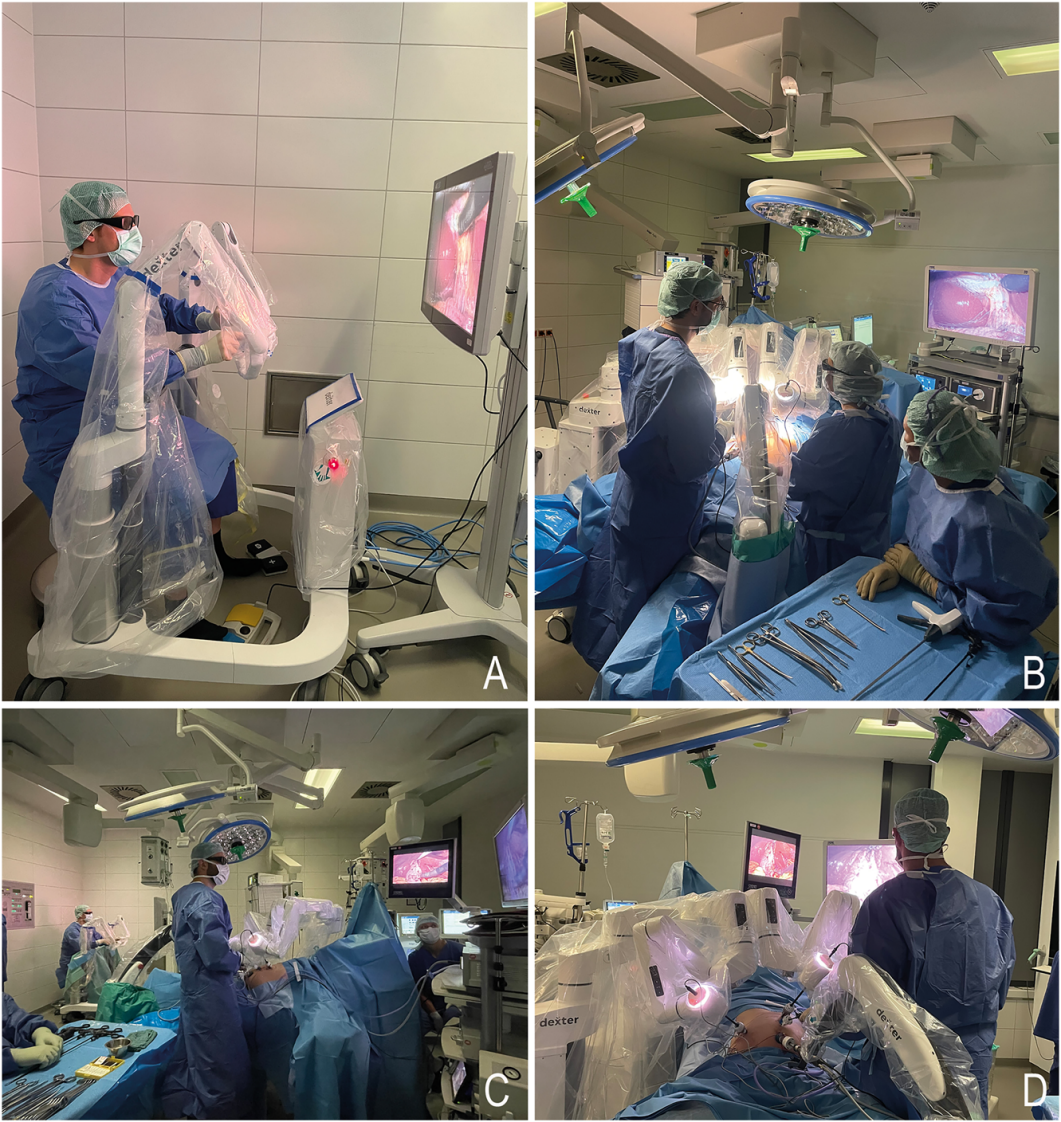
Intraoperative views of the Dexter^®^ robot surgery (A) Surgeon at the console (B) view from the nursing department (C) assisting surgeon from the rear (D) foot-end view of the patient.

### Surgical procedure

– **Preoperative Preparation:** Patients were prepared according to standard preoperative protocols. Preoperative imaging studies were reviewed to plan port placement and surgical strategy.
– **Port Placement:** The ports were strategically placed to optimize access to the surgical field while minimizing interference with the robotic arms. Typically, four trocars were placed, consisting of one 5 mm, two 10 mm, and one 12 mm access.
– **Robotic Assistance:** The second surgeon at the operating table assisted with gallbladder retraction, clipping, and instrument exchange, while the leading surgeon at the console performed the preparation and actual cholecystectomy. The Dexter robot was utilized for key steps of the cholecystectomy, including dissection of Calot’s triangle, preparation and division of the cystic duct and artery, and dissection of the gallbladder from the liver bed. The robot’s enhanced visualization was particularly beneficial during these critical steps. Intraoperative impressions are shown in Figure 3.
– **Completion of Surgery:** After robotic dissection, the gallbladder was removed through one of the ports, and the surgical field was inspected for hemostasis. Ports were then closed, and the patient was transferred to the recovery room.

### Statistics

Statistical analyses were performed using SPSS version 29 (IBM SPSS, Chicago, IL, USA) and Microsoft Excel (Version 16, Redmond, WA, USA). Continuous variables were summarized as mean ± standard deviation (SD). The unpaired t-test was utilized to compare the means between two independent groups. A p–value ≤0.05 was considered to indicate statistical significance. The large language model ChatGPT 4 (OpenAI, San Francisco, USA, RRID:SCR_023775) was used to improve the language of the manuscript. After using, the authors reviewed the content and take full responsibility for the published article.

## Results

### Implementation metrics

The implementation process included extensive training for a total of six surgeons and thirty nurses. Surgeon training involved a minimum of 20 h of simulation training, followed by a minimum of five supervised procedures before they were entitled to perform an operation as a leading surgeon. Nursing and technical staff training included theoretical and practical hands-on sessions.

### Patient characteristics

The patient cohort consisted of individuals with a mean age of 49.2 ± 12.2 years (range: 25–74 years) and BMI of 28.1 ± 4.0 kg/m^2^ (range: 21.6–36.3 kg/m^2^). The male-to-female ratio was 7:13. The majority of patients were classified as ASA class I (n=4; 20 %) or II (n=14; 70 %), indicating low to moderate surgical risk. The vast majority of patients (n=18, 90 %) presented with symptomatic cholelithiasis or cholecystitis. In two other cases, the surgery was indicated due to gallbladder polyps≥1 cm. Preoperative imaging confirmed the presence of gallstones in 18 patients (90 %). Eleven patients (55 %) had undergone previous abdominal surgeries. The surgical history included appendectomies (n=4; 20 %), cesarean sections (n=6; 30 %), inguinal hernia repairs (n=2; 10 %) and myome enucleation (n=1; 5 %) (Table 2).

**Table 2:** Patient demographics and clinical characteristics.

Demographics	Patients (n=20)
Age (mean ± SD)	49.2 ± 12.2 years
Gender	7 ♂: 13 ♀
ASA class (I; II; III)	I=4; II=14; III=2
BMI (mean ± SD)	28.1 ± 4.0 kg/m^2^
Preoperative diagnosis	
– Symptomatic cholelithiasis	18/20 (90 %)
– Gallbladder polyps	2/20 (10 %)
Previous surgeries (total)	11/20 (55 %)
– Appendectomy	4/20 (20 %)
– Caesarean section	6/20 (30 %)
– Inguinal hernia	2/20 (10 %)
– Myoma enucleation	1/20 (5 %)

### Operative metrics and postoperative outcomes

The preoperative preparation time of the OR nursing team decreased from an average of 63.0 ± 23.9 min in the first 10 procedures to 50.7 ± 11.8 min in the second 10 surgeries (Table 3). Draping of the Dexter^®^ robot improved significantly from 14.0 ± 2.1 min to 11.6 ± 1.6 min (p=0.0142).

**Table 3:** Operative metrics, including nursing staff OR setup, robotic arm draping, operation phases, and perioperative hemoglobin reduction.

Procedure time (mean ± SD)	Cases 1–10	Cases 11–20	p-Value
Nursing staff preparation time	63.0 ± 23.9 min	50.7 ± 11.8 min	not significant
Robot draping time	14.0 ± 2.1 min	11.6 ± 1.6 min	*p=0.0142*
Operation time	130.5 ± 25.7 min	99.7 ± 21.8 min	*p=0.0134*
Docking time	11.4 ± 4.1 min	7.1 ± 2.1 min	*p=0.0144*
Surgeons console time	48.8 ± 24.7 min	44.1 ± 29.0 min	not significant
Perioperative hemoglobin decrease	1.2 ± 0.9 g/dl	1.1 ± 1.0 g/dl	not significant

Due to the increasing routine of the surgical team, the average setup time (“docking time”) for the robotic system also decreased significantly from 11.4 ± 4.1 min during the first 10 surgeries to 7.1 ± 2.1 min (p=0.0144).

The mean effective operation time (incision to suture time), improved significantly from 130.5 ± 25.7 min to 99.7 ± 21.8 min (p=0.0134), which demonstrates iterative improvements. During this time, the surgeon spent an average of 48.8 ± 24.7 min (case 1–10) respectively 44.1 ± 29.9 min (case 11–20) in the console.

The mean estimated blood loss was 19.5 ± 31.4 mL (range: 10–150 mL) (Table 4). There were no intraoperative adverse events and no conversion to either fully laparoscopic or open surgery in the patients included.

**Table 4:** Surgical outcomes and postoperative complications according to Clavien–Dindo classification.

Surgical outcomes	Parameters (n=20)
Blood loss (mean ± SD)	19.5 ± 31.4 mL
Length of hospital stay (mean ± SD)	3.1 ± 1.4 days
Conversion to open surgery, %	0 % (n=0)
**Postoperative complications,** **%**	10 % (n=2)
*Minor complications (Grade I-IIIa)*	5 % (n=1)
– Superficial wound infection	5 % (n=1)
– Hematoma/seroma	0 % (n=0)
– Minor biliary leakage	0 % (n=0)
– Abscess	0 % (n=0)
– Minor bleeding	0 % (n=0)
*Major complications (Grade IIIb)*	5 % (n=1)
– Postoperative hemorrhage	5 % (n=1)
– Major biliary leakage	0 % (n=0)
– Clipping of liver artery/common bile duct	0 % (n=0)

Within 30 days postoperatively, there were no device-related complications. One grade IIIb event according to the Clavien–Dindo classification was documented. This involved a postoperative hemorrhage on the day of surgery from an artery of the greater momentum after adhesiolysis, which necessitated laparoscopic surgical re-intervention. Furthermore, one uncomplicated superficial wound infection occurred at a trocar-site. The mean length of hospital stay was 3.1 ± 1.4 days (range: 2–8 days). The extended hospital stay of eight days was due to the aforementioned reoperation including hematoma evacuation and hemostasis.

**Figure 4:**
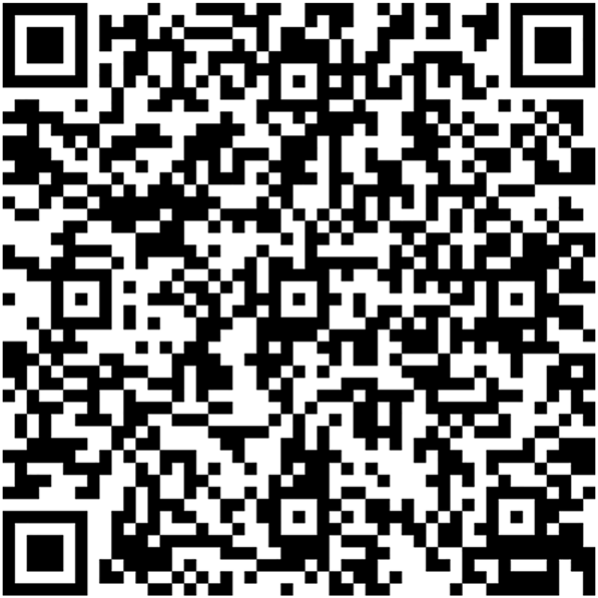
Video clip of cholecystectomy using the dexter robotic system (2 min). To access the video, please scan the QR code or use the following link: https://ukbcloud.uniklinik-bonn.de/public/download-shares/i2995lqKDMu9gSduQ4iLGp1dNSkZyOYm.

A short video summary of a Dexter^®^ cholecystectomy, recorded during this study, can be viewed by scanning the QR code (Figure 4).

### Postoperative survey

A postoperative survey was conducted 30 days following surgery via telephone to evaluate patient outcomes. The survey included a structured questionnaire assessing postoperative pain, time to complete recovery, satisfaction with cosmetic results and overall satisfaction. Out of the 20 patients, 13 were successfully reached and participated in the survey. These patients reported a mean pain score of 2.3 ± 1.2 (scale 1–10, 1 is the lowest pain) one week after surgery and an average time to full recovery of 2.5 ± 1.3 weeks (Table 5). Most patients indicated a rapid return to normal activities. The mean satisfaction score for cosmetic results was 1.2 ± 0.4 (1 indicating “very satisfied”) and the mean overall satisfaction score was 1.3 ± 0.6. These results reflect a high degree of satisfaction with the minimally invasive robotic surgery, characterized by low postoperative pain, swift recovery and favorable cosmetic outcomes, thereby demonstrating a high level of acceptance of the robotic approach.

**Table 5:** Telephone survey results. Pain score is rated on a scale from 1 (least pain) to 10 (worst pain). Satisfaction scores are categorized as follows: 1 for very satisfied, 2 for satisfied, 3 for moderately satisfied, 4 for not very satisfied, and 5 for not satisfied.

Survey metrics (mean ± SD)	Mean rating (n=13)
Pain one week after surgery	2.3 ± 1.2
Time to full recovery in weeks	2.5 ± 1.3
Satisfaction with cosmetic result	1.2 ± 0.4
Overall satisfaction	1.3 ± 0.6

## Discussion

Our experience with the Dexter surgical robot underscores the critical importance of a well-structured and methodical implementation process in compliance with the IDEAL framework.

The value of a structured pathway, such as IDEAL, for the implementation and evaluation of surgical robots was recently reinforced by an interdisciplinary consensus panel [7]. However, the actual situation regarding the use of robotic technology in cholecystectomies differs considerably from this approach [14].

Following the initial first-in-human publications of the Dexter^®^ surgical robot [19], 23], we now present the first investigation encompassing IDEAL stages 2a and 2 b. After a detailed description of the procedural aspects, and in accordance with the IDEAL recommendations, we placed particular emphasis on the transparent presentation of the training phase and workflow optimization process, with the aim of demonstrating the learning effects.

The training program was designed to ensure that all team members, including surgeons and nurses were proficient in the use of the Dexter robot. This training included cadaveric training and dry lab training over a period of four months, which was essential to mitigate the initial learning curve and enhance the team’s confidence and efficiency in performing robotic-assisted procedures. Studies, conducted in this field, support the view that thorough training and simulation significantly improve surgical outcomes and reduce operative times, as the team becomes more experienced [24], 25].

The development and refinement of the surgical workflow constituted another critical aspect of our implementation process. By establishing clear protocols for preoperative planning, intraoperative management (including emergency scenarios), and postoperative care we ensured that all team members were aware of their respective roles and responsibilities, which is crucial for maintaining efficiency and patient safety. The importance of well-defined protocols and team coordination in robotic surgery has been highlighted in numerous studies [26], 27], thereby supporting our approach.

The implementation of continuous monitoring and data collection, coupled with regular evaluation of the team’s communication, were pivotal in tracking progress and making data-driven adjustments to our protocols. However, it is recommended that a clinical outcomes-based approach be adopted as the primary assessment focus at the earliest possible stage [7]. Our monitoring system provided valuable insights into operative metrics and patient outcomes. Meticulous documentation was conducted by study nurses and graduate students to ensure data accuracy and completeness.

In terms of clinical outcomes, the operative times observed in this study were moderately longer than those reported for conventional laparoscopic cholecystectomy [28]. Notably, the only published study to date on robotic-assisted cholecystectomies using the Dexter^®^ robot, conducted by Conrad et al. reported a slightly shorter mean operative time of 58 min for 15 procedures [23]. Docking times were similar in both studies, with Conrad et al. reporting an average of 6.5 min.

The data from their initial 11 robotic cholecystectomies conducted with the Dexter^®^ surgical robot were presented by Hotz et al. at the Swiss College of Surgeons Annual Meeting 2023. The operating time of 99 min was comparable to that observed in our study, whereas the console time of 55 min was slightly longer than that recorded in our study (only the congress abstract has been published [29]).

As our clinic is a level 3 maximum care hospital, there were a number of pre-existing conditions in the patient population that made the procedures more difficult. These included, for example, patients with PSC (primary sclerosing cholangitis), patients with a condition following ERCP and stent placement, purulent cholangitis, fibrosing cholecystitis and several obese patients (grade I and II obesity). These factors could explain the slight longer average duration of surgery.

In the context of the IDEAL framework, an investigation of the safety and effectiveness outcomes of robotic-assisted procedures is recommended. The minimal blood loss, low complication rates, and high patient satisfaction demonstrated in our study are indicative of the overall safety and efficacy of the Dexter^®^ surgical robot. Consistent with reports in the literature, the trade-off is a longer operating time and higher costs [30], [31], [32].

Although laparoscopic cholecystectomy has become a common outpatient procedure in many countries worldwide, in Germany it is still generally performed on an inpatient basis. The mean length of stay of 3.1 days observed in our study is comparable to that of a laparoscopic cholecystectomy [33].

It is evident that the cost-effectiveness of the surgical robots like the Dexter^®^ remains a topic of discussion and requires further evaluation [34]. The financial aspects of robotic surgery present significant challenges, particularly in regard to reimbursement. As an example of such challenges, in Germany, the absence of reimbursement for robotic surgery represents a significant obstacle, affecting the economic feasibility and wider adoption of this technology [35], 36].

The environmental impact of robotic surgery is considerable. A comparative analysis revealed that the carbon footprint is 38 % higher in comparison to laparoscopy [37]. It is notable, that the Dexter^®^ is one of the few surgical robots, that utilizes disposable single-use instruments [38], a practice that could be improved upon in terms of sustainability.

Our findings indicate that the Dexter robot significantly enhanced surgical precision and efficiency. The minimal blood loss and avoidance of conversion to laparcopic or open surgery highlight the robot’s effectiveness in performing delicate dissections and maintaining hemostasis.

While our implementation process was successful, it is important to acknowledge the limitations of our study. The extended operative times observed during the initial phase reflect the learning curve associated with the new technology. However, as the surgical team becomes more experienced, we anticipate a reduction in these times, aligning with the performance of traditional laparoscopic procedures. Future studies should focus on long-term outcomes and the scalability of our implementation framework across different surgical departments and institutions. Furthermore an assessment study (stage 3 of the IDEAL framework) comparing the Dexter^®^ with the current standard in robotic surgery, the Intuitive Da Vinci^®^, would be a valuable and beneficial next step.

In conclusion, the IDEAL-compliant implementation of the Dexter surgical robot in our clinic has significantly advanced our surgical practice. This study highlights the critical role of meticulous planning, training, and workflow integration in the successful adoption of robotic systems. Our interim analysis serves as a milestone, and we plan to continue with advanced implementation phases. As technological advancements continue to reshape surgical practice, an expertise in robotic implementation positions teams to succeed in this transformative field.
